# A case report of intramural pregnancy with uterine rupture: a warning signal from ineffective evacuation

**DOI:** 10.3389/fmed.2026.1778238

**Published:** 2026-02-13

**Authors:** Danlin Wang, Huawei Lei, Qiufen Lu, Shaoxia Yan, Xiang Fu, Yan Fang

**Affiliations:** Department of Gynaecology, Tongren People's Hospital, Tongren, Guizhou, China

**Keywords:** emergency laparotomy, ineffective evacuation, intramural pregnancy, pregnancy with IUD, uterine rupture

## Abstract

**Objective:**

Intramural pregnancy is a rare form of ectopic pregnancy. We report a case of undiagnosed intramural pregnancy that progressed from ineffective evacuation to uterine rupture.

**Patient:**

A challenging case of a 41-year-old woman who experienced uterine rupture at 15 weeks’ gestation due to intramural pregnancy.

**Results:**

The patient was once misdiagnosed with an intrauterine pregnancy with an intrauterine device (IUD) and underwent ineffective evacuation. After 9 weeks, she was referred to our hospital with complaints of sudden-onset lower abdominal distension. An emergency ultrasound revealed a fetus without cardiac activity floating in the peritoneal cavity and a clear rupture site at the uterine fundus. Given the clinical suspicion of uterine rupture, an emergency laparotomy was performed. Intraoperatively, the peritoneal cavity contained approximately 1700 mL of hemoperitoneum. The amniotic sac and fetus were found to be free in the abdominal cavity. Notably, a 3.5 × 3.0 cm defect was found at the uterine fundus with active bleeding, which did not communicate with the uterine cavity. The bilateral fallopian tubes and ovaries appeared grossly normal. After the intraperitoneal blood was evacuated, the dead fetus along with the amniotic sac was removed. The uterus was repaired with a continuous absorbable suture. The estimated blood loss was 1800 mL. Finally, the patient recovered well and was discharged.

**Conclusion:**

We present a case of an undiagnosed intramural pregnancy that progressed from ineffective evacuation to uterine rupture. An emergency laparotomy was performed to remove the dead fetus and repair the uterine defect. Therefore, intramural pregnancy should be strongly considered in women with previous uterine trauma, who present with persistent pregnancies with IUDs and a history of ineffective evacuation.

## Introduction

Intramural pregnancy is a rare form of ectopic gestation in which the embryo is implanted within the myometrium, outside the endometrial cavity, and accounts for less than 1% of all ectopic pregnancies (1). This condition is often associated with prior uterine trauma, including that caused by cesarean section, or myomectomy, and other forms of myometrial damage (2).

Owing to its nonspecific early symptoms and imaging challenges, intramural pregnancy is notoriously difficult to diagnose. Early transvaginal sonography frequently misinterprets an intramural pregnancy as a normally implanted intrauterine gestation, leading to delayed diagnosis until severe complications, such as uterine rupture, occur (3). As gestational age increases, the expanding gestational sac within the myometrium progressively thins and weakens the uterine wall, substantially increasing the risk of rupture. This condition can lead to catastrophic outcomes, including massive intra-abdominal hemorrhage, hemodynamic instability, and even maternal mortality (4).

Given the diagnostic challenges and life-threatening potential, heightened clinical suspicion and advanced imaging modalities are crucial for timely intervention. Expectant management may be considered a first-line strategy in carefully selected asymptomatic patients with spontaneous resolution (5). However, management typically requires emergent surgical intervention once rupture occurs, underscoring the importance of early detection to preserve fertility and prevent mortality (6). In this study, we present a case of undiagnosed intramural pregnancy that progressed from ineffective evacuation to uterine rupture, highlighting that intramural pregnancy should be suspected in women with a history of repeated uterine trauma when pregnancy persists despite attempted evacuation.

## Case presentation

**Figure 1:**
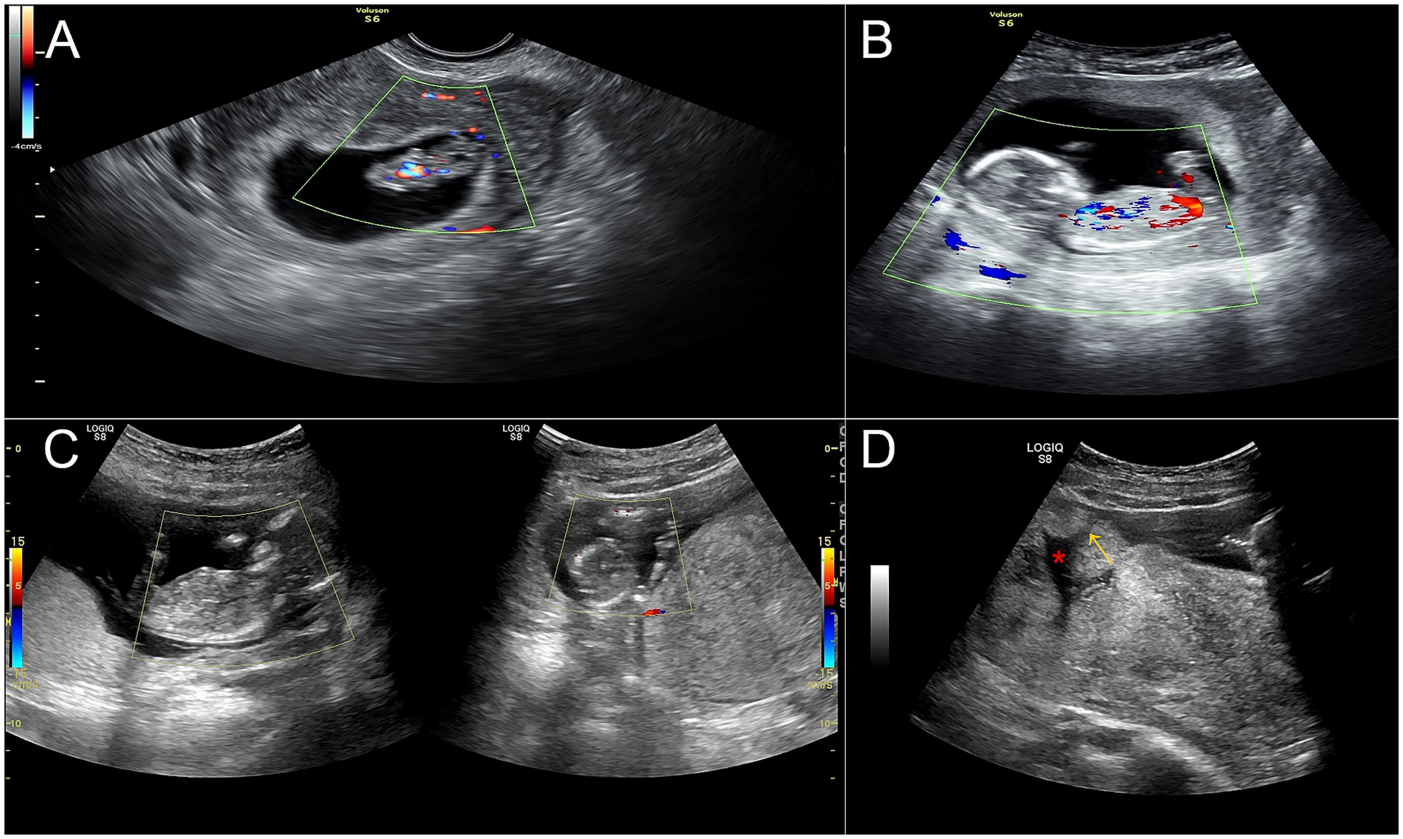
Ultrasound revealed the clinical course from ineffective evacuation to uterine rupture. **(A)** A continuing pregnancy after suction evacuation with a fetal pole measuring approximately 25 mm. **(B)** A 15-week fetus before uterine rupture. **(C)** A fetus without cardiac activity floating in the peritoneal cavity. **(D)** Myometrial discontinuity was noted at the uterine fundus, with a clear defect (red asterisk) and rupture site (yellow arrow).

This report presents a challenging case of a 41-year-old woman who experienced uterine rupture at 15 weeks’ gestation due to an intramural pregnancy. The patient was gravida 9, para 4, with four living children. She had two cesarean sections and two spontaneous vaginal deliveries, as well as four induced abortions performed by uterine curettage during the first trimester. Her last menstrual period was on October 20, 2021, and the estimated date of delivery was August 27, 2022. Her pregnancy test was positive and transvaginal ultrasound confirmed an intrauterine pregnancy with an intrauterine device (IUD) on November 28, 2021. Given the increased risks of complications, she underwent IUD removal and suction evacuation for the unintended pregnancy on December 4, 2021. However, a follow-up ultrasound on January 1, 2022 unexpectedly revealed a continuing intrauterine pregnancy with a fetal pole measuring approximately 25 mm (Figure 1A). She decided to continue the pregnancy, but prenatal care remained irregular thereafter.

On February 5, 2022, she was referred to a local hospital with complaints of sudden-onset lower abdominal distension. Ultrasound revealed an intrauterine pregnancy with a hypoechoic lesion near the appendix, prompting a provisional diagnosis of appendicitis during pregnancy (Figure 1B). Therefore, she was urgently referred to our emergency department. Within 3 h, her pain worsened significantly and was accompanied by dizziness and cold, clammy extremities. Physical examination revealed a blood pressure of 95/67 mmHg, diffuse abdominal tenderness, and a poorly defined uterine contour. The hemoglobin concentration was 98 g/L. An emergency ultrasound revealed that a fetus without cardiac activity was floating in the peritoneal cavity (Figure 1C). In addition, myometrial discontinuity was noted at the uterine fundus, with a clear rupture site (Figure 1D).

**Figure 2:**
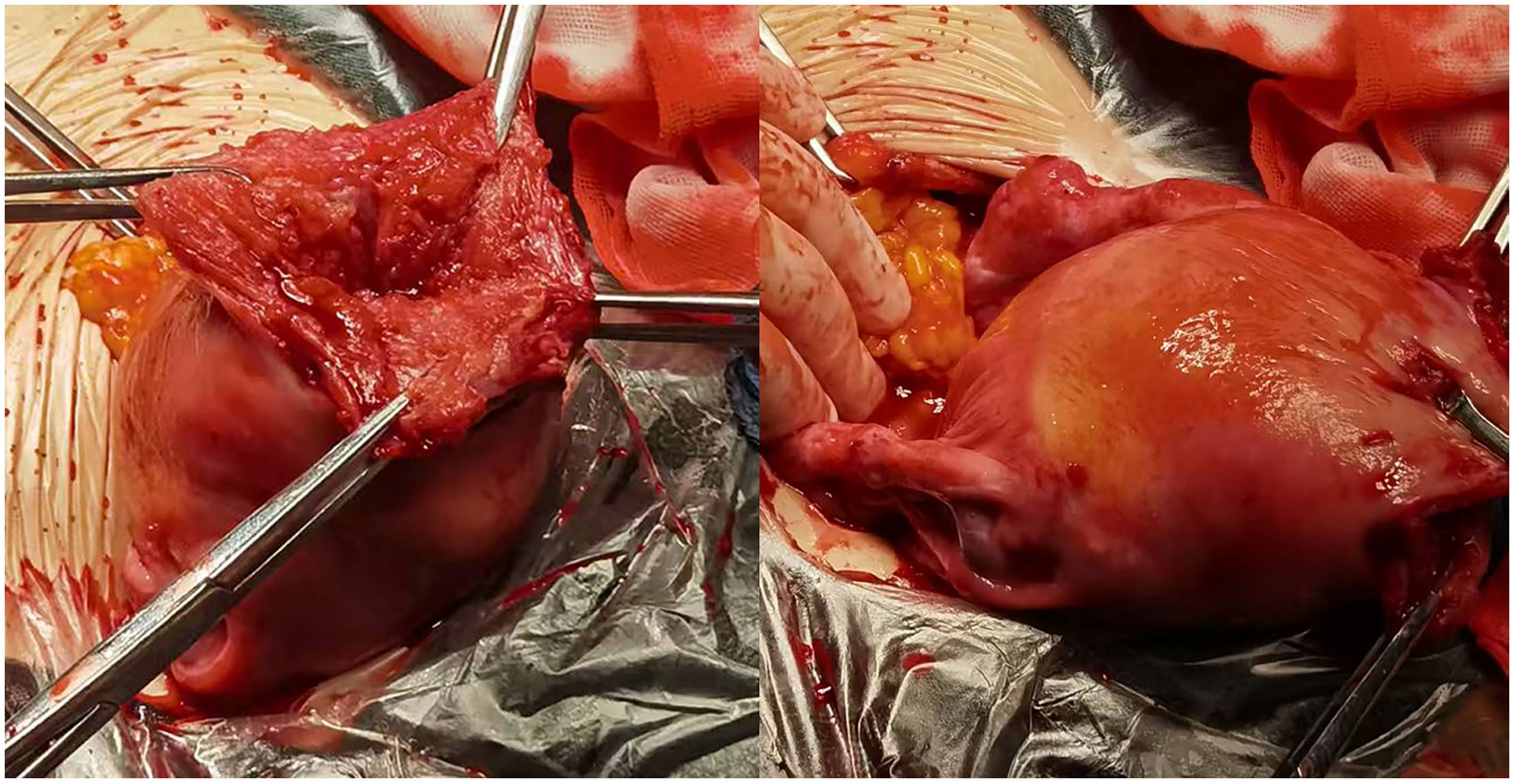
Intraoperatively, a 3.5 × 3.0 cm defect was found at the uterine fundus with active bleeding, which did not communicate with the uterine cavity.

After informed consent was obtained, an emergency laparotomy was performed. Intraoperatively, the peritoneal cavity contained approximately 1700 mL of hemoperitoneum. The amniotic sac and fetus were found to be free in the abdominal cavity. Notably, a 3.5 × 3.0 cm defect was found at the uterine fundus with active bleeding, which did not communicate with the uterine cavity (Figure 2). The bilateral fallopian tubes and ovaries appeared grossly normal. Therefore, intramural pregnancy with uterine rupture was diagnosed on the basis of the above findings. After the intraperitoneal blood was evacuated, the dead fetus and the amniotic sac were removed. The uterine defect was repaired with a continuous absorbable suture. The estimated blood loss was 1800 mL The serum *β*-human chorionic gonadotropin (β-hCG) concentration was 2,066 mIU/ml 2 days after the operation. The patient recovered well and was discharged on February 10, 2022. At her outpatient follow-up after 2 weeks, the serum β-hCG concentration was 17.76 mIU/mL, and ultrasound revealed a normal-sized uterus. Appropriate written consent for publication was obtained.

## Discussion

In this study, we report a case of an undiagnosed intramural pregnancy that progressed from ineffective evacuation to uterine rupture. Intramural pregnancy is a rare form of ectopic pregnancy defined by a gestation located entirely within the uterine wall and completely surrounded by the myometrium (1). Intramural pregnancy is typically diagnosed via transvaginal ultrasound, which reveals a gestational sac embedded within the myometrium, without a decidual reaction near the trophoblastic tissue. The final diagnosis was confirmed by histological examination, which revealed chorionic villi and trophoblastic cells embedded within the myometrium (3). Owing to its diagnostic complexity and catastrophic outcomes, early recognition through increased clinical awareness and advanced imaging is essential to enable timely intervention.

Prior uterine trauma, such as that caused by cesarean section, myomectomy, and induced abortions, is a well-recognized risk factor for intramural pregnancy. Such risk factors may disrupt the integrity of the endometrial–myometrial interface, facilitating direct blastocyst invasion and subsequent implantation within the myometrium (7). The studied patient had a history of 2 cesarean deliveries and 4 induced abortions, and an IUD was inserted in 2019. However, she was misdiagnosed with an intrauterine pregnancy with an IUD. Although the IUD is an effective approach for reversible contraception, pregnancy may rarely occur during use. A nationwide study reported an overall pregnancy rate of 0.02% among IUD users (8). In addition, IUD use is recognized as an independent risk factor for ectopic pregnancy (9). For example, users of the levonorgestrel IUD had a 2.6-fold higher risk of ectopic pregnancy (10). In this situation, the presence of an IUD may interfere with endometrial implantation, thereby leaving the embryo to implant at an extrauterine site (11). Therefore, ectopic pregnancy should be a primary consideration in the initial evaluation of IUD-related pregnancies. However, ectopic pregnancy was not suspected in this patient, leading to delayed diagnosis.

Given the increased risks associated with an IUD-related pregnancy, such as spontaneous abortion, chorioamnionitis, and ectopic pregnancy (5, 19), and the patient’s intention to terminate the pregnancy, she opted to und-ergo IUD removal and suction evacuation at an outside facility. However, follow-up ultrasound revealed a continuing intrauterine pregnancy. In South Africa, 1.1% of women who have undergone surgical abortion experience incomplete or failed procedures (12). In such cases, immediate inspection of the fresh tissue aspirate after surgical abortion is critical. In addition, the absence of chorionic villi should raise suspicion for ectopic implantation and can facilitate early diagnosis (13). When pregnancy persists during follow-up, whether confirmed by ultrasonography or pregnancy tests, patients are typically offered a second procedure. However, the reported patient decided to continue the pregnancy. In such circumstances, the persistence of pregnancy with an IUD and ineffective evacuation should strongly prompt consideration of an abnormal implantation site.

Notably, the absence of classic warning signs, such as vaginal bleeding or severe abdominal pain during early gestation, can contribute to misdiagnosis, allowing intramural pregnancy to progress undetected until catastrophic complications such as uterine rupture occur (14). Although nearly half of published cases involve diagnosis by ultrasound, the condition remains frequently underdiagnosed in routine clinical practice because of its nonspecific sonographic features and extreme rarity (15). Typical ultrasound findings may mimic an intrauterine or interstitial pregnancy when the gestational sac is deeply embedded within the myometrium without disruption of the endometrial cavity. In addition, magnetic resonance imaging (MRI) serves as a valuable adjunct to clearly delineate the spatial relationship between the gestational sac and the endometrial cavity, thereby assisting in definitive diagnosis and guiding management (16).

Despite advances in obstetric care, uterine rupture remains a life-threatening emergency, often culminating in hemorrhagic shock and permanent infertility (17). Approximately 62% of women with uterine rupture had a history of uterine surgery, such as cesarean delivery or dilation and curettage (4). In the context of intramural pregnancy, the risk increases significantly. The ever-expanding gestational sac exerts progressive pulling force on the thinning uterine wall, ultimately leading to catastrophic uterine rupture. Moreover, a gestational age greater than 10 weeks is a well-documented risk factor for both uterine rupture and subsequent hysterectomy. In addition, implantation in the uterine fundus appears to confer particularly high risk (1). Once rupture occurs with hemodynamic instability, emergency hysterectomy is often one of the common means to control hemorrhage. With respect to second-trimester uterine rupture during intramural pregnancy, hysterectomy rates can reach as high as 14.3% (18).

However, early diagnosis of intramural pregnancy before rupture can lead to the development of fertility-sparing strategies, including expectant management, methotrexate administration, or conservative surgical enucleation (5). In this case, despite presenting with acute rupture at 15 weeks’ gestation, urgent laparotomy enabled uterine preservation through meticulous hemostasis and layered myometrial repair. These outcomes suggest that timely recognition of intramural pregnancy is paramount. It not only permits conservative treatment but also safeguards future reproductive potential and prevents maternal mortality.

## Conclusion

Intramural pregnancy is a rare form of ectopic pregnancy. In this study, we present a case of an undiagnosed intramural pregnancy that progressed from ineffective evacuation to uterine rupture. The persistence of pregnancy with an IUD and ineffective evacuation should strongly prompt consideration of ectopic pregnancy. Early detection is crucial for preserving fertility and preventing mortality. In particular, intramural pregnancy should be strongly considered in women with previous uterine trauma, who present with persistent pregnancies with IUDs and ineffective evacuation.

## Data Availability

The original contributions presented in the study are included in the article/supplementary material, further inquiries can be directed to the corresponding author.
